# Parents and newborn “togetherness” after birth

**DOI:** 10.1080/17482631.2022.2026281

**Published:** 2022-01-24

**Authors:** Katarina Patriksson, Lotta Selin

**Affiliations:** a Department of Health Sciences, University West, Trollhättan, Sweden; b Division of Paediatrics, NU-Hospital Group, Trollhättan, Sweden; c Institute of Health and Care Sciences, University of Gothenburg, Gothenburg, Sweden; d Department of Obstetrics and Gynecology, NU-Hospital Group, Trollhättan, Sweden

**Keywords:** Caregivers, newborn, parents, skin-to-skin contact, zero separation

## Abstract

**Purpose:**

Zero separation is a family-centred approach where newborns should be accompanied by their parents, regardless of the type of birth or health status. To our knowledge, few studies have described the way this approach is realized in clinical practice. This study describes situations of separation between mother/partner and newborn after birth on the labour ward, maternity ward and at the neonatal unit.

**Method:**

An observation study was conducted during four months at a Swedish hospital. All caregivers at the three units were given the task of collecting the data. A semantic thematic analysis was performed with an inductive approach.

**Results:**

Six themes emerged from the analysis. Two themes were common to all three units, one theme was common to two units and three themes emerged at only one unit. The themes describe various causes of separation, such as organizational and economic barriers, clinical routines, parents’ own decisions, shortage of collaboration within and between units, as well as a shortage of interprofessional communication.

**Conclusion:**

Our study shows that there is still a gap between the latest evidence-based knowledge of the importance of zero separation and current practice in newborn care. There is a need for continuous collaboration between all units responsible for the care of mother and newborn.

## Introduction

The first hour of life is an important and meaningful period and, in the literature, it is often referred to as the “golden hour”, “sacred hour” or “magic hour”.

At the moment of birth and during the hours and days that follow, mothers and newborns have a physiological need to be together. Keeping mothers and newborns together is a safe and healthy birth practice (Bergman, 2014; Crenshaw, 2014; Moore et al., 2016). Skin-to-skin contact refers to the contact between a newborn and a parent’s bare chest when the newborn is placed in a prone position, naked and with towels covering its back (Anderson et al., 2007; Crenshaw, 2014). It is one of the main components of kangaroo mother care (KMC), a strategy that has been developed to care for premature infants born before 37 weeks of gestational age or infants with a low birth weight up to 2,500 g (Charpak et al., 2021). Skin-to-skin contact is recommended for all women and newborns, regardless of the type of birth or newborn feeding method. These recommendations state that, for all stable women and their newborns, skin-to-skin contact should begin immediately after birth (World Health Organization [WHO] and United Nations Children’s Fund, 2009) and it is regarded as the first part of maternal-infant “togetherness” (Bergman & Bergman, 2013).

When the newborn is placed skin to skin with the mother, there is a significant increase in oxytocin, which will reduce maternal and newborn stress and help the newborn transition to

postnatal life (Bergman & Bergman, 2013; Buckley, 2015; Moore et al., 2016; Uvnäs-Moberg et al., 2015). Furthermore, skin-to-skin contact with the mother will help to meet the newborn’s basic biological needs, activate neuroprotective mechanisms, enable early neurobehavioural self-regulation and give the newborn a more stable heart rate, blood pressure, breathing and higher blood glucose (Buckley, 2015; Moore et al., 2016; Widström et al., 2010). Skin-to-skin contact after birth has additional benefits, including increased infant axillary temperature and extended exclusive breastfeeding after hospital discharge (Moore et al., 2016). Women who experience skin-to-skin contact and breastfeeding after birth are two times less likely to have a postpartum haemorrhage than women who do not experience skin-to-skin contact immediately after birth (Saxton et al., 2015).

Skin-to-skin contact and the release of oxytocin by the mothers will promote maternal-newborn attachment (Bergman & Bergman, 2013; Buckley, 2015; Moore et al., ; Uvnäs-Moberg et al., 2015). In a study by Noren et al., mothers were asked about their experience of skin-to-skin contact with their newborns. The mothers believed that skin-to-skin contact had a positive impact on their newborns; they both slept better and were calmer during skin-to-skin contact (Norén et al., 2018). Holding skin to skin and touching and smelling their newborns gave the parents an increased feeling of closeness of love and attachment and was the first time they felt their infant was theirs (Flacking et al., 2016). A dose response to the longer duration of initial skin-to-skin contact has been found positively to affect maternal sensitivity, dyadic mutuality and reciprocity at one year postpartum (Bystrova et al., 2009). The importance of early maternal-infant skin-to-skin contact, as well as contact as often as possible and for as long as possible, for at least the duration of the postpartum stay, has been specifically highlighted (Bramson et al., 2010).

Zero separation is a concept introduced by Bergman where zero separation is defined as skin-to-skin contact with one parent or one of the parents being present with the newborn all the time (Bergman, 2014). The Swedish National Board of Health and Welfare defines zero separation as couplet care between the mother and the newborn, where zero separation is an important part of family-centred care. In this case, couplet care means that, even if the mother requires medical care, she should be allowed to be cared for together with her newborn (Swedish National Board of Health and Welfare, 2020). According to the Global Alliance for Newborn Care, zero separation is a family-centred approach where the newborns should be accompanied by their parent/parents, regardless of whether or not they have health problems (Global Alliance for Newborn Care, 2020).

In Sweden, skin-to-skin contact follows the guidelines from the World Health Organization (WHO, 2003). A survey conducted by the Swedish National Board of Health and Welfare has shown that there are large variations in and limitations to the way mother and newborn in neonatal care are cared for together (Swedish National Board of Health and Welfare, 2017). Furthermore, there is a lack of knowledge of how skin-to-skin contact is implemented in clinical practice for all newborns, regardless of the type of birth or health status. The aim of this study was therefore to describe situations of separation between the mother/partner and newborn after birth on a labour ward, maternity ward and at a neonatal unit. In addition, the aim was to describe the context in which these separations arose.

In this paper, the father is referred to as “the partner” or “the parent”.

## Materials and methods

### Design

A project was initiated with the aim of reducing separation between mother/partner and newborn by improving working methods and routines within and between units involved in the care of both the pregnant woman and the mother and newborn after birth.

This study describes the first phase of the project in which situations of separation between mother/partner and newborn during the care after birth were observed. Furthermore, based on these observations, the need for collaboration within and between units involved in the care is described.

### Settings

The study was conducted on the labour ward, maternity ward and at the neonatal unit at NU Hospital Group, situated in the western part of Sweden.

The labour ward has around 3,600 labours a year and all labours from 28 gestational weeks were included in the study. After a normal delivery, the mother stays for postpartum care for approximately two hours on the labour ward before transferring to the maternity unit.

The maternity unit has 19 single rooms and four rooms with two beds and, in 2019, the average number of days of inpatient care was 3.1 for primipara and 1.9 for multipara women. A mother and newborn in good health might leave the maternity ward after six to eight hours.

In general, first-time parents can stay together on the maternity ward in a single room. For parents with more children, the mother often stays alone with the newborn and shares a room with another mother.

The neonatal units in Sweden are divided into three levels of care. Level I is basic neonatal care and Level II is specialist neonatal care. Level III is the subspeciality of neonatal intensive care and is subdivided into A-D, where D is full intensive care for extremely preterm newborns (Stark, 2004). The neonatal unit at NU Hospital Group belongs to Level II and has 16 beds in different rooms for care. There are nine family rooms at the unit where both parents can stay. The condition of the newborn determines whether it is together with its parents in a family room all the time, part of the time or not at all. In 2019, the average number of days of inpatient care was 6.9 days.

### Data collection

Data were collected during a period from March to June in 2019.

All caregivers at the three units (midwives, paediatric nurses, nurses, physicians, nurse assistants) were given the task of collecting the data, i.e., situations of separation between mother/partner and newborn. Prior to the data collection, a team of members from each unit was set up and several meetings with all caregivers were organized. At these meetings, information, and a deeper understanding of the importance of skin-to-skin contact and the correct definition of zero separation were given, as well as information about the protocol for data collection. Regular meetings were organized between the team members. A team of members from each unit was organized with the aim of supporting other caregivers to observe, even at times when there was a heavy workload.

A protocol common to all the units was designed and used for each observation. If a separation between mother/partner and newborn was observed by the caregivers during care after birth and at the hospital stay. These situations were then documented in the protocol in the caregivers’ own words in a narrative way. The whole data material consisted of free-text material. The observations were made on all working shifts (day/evening/night). During the four months, 197 observations were collected, 54 observations from the labour ward, 103 observations from the maternity ward, while 40 observations were collected from the neonatal ward. The caregivers simply observed the situations of separation, without interfering or directing the action in any way.

### Data analysis

A semantic thematic analysis was performed using an inductive approach (Braun & Clarke, 2006). Both authors worked together and were responsible for and contributed to the analysis. The data represented the observed situations and were analysed separately for each unit. The analyses described by Braun and Clarke (2013) contains six non-linear phases, familiarization with the data, coding, searching for themes, reviewing the themes, defining and naming the themes and writing up.

The familiarization was made by careful reading and re-reading, in order to reach an understanding of the wholeness of the data set. Codes were identified using data-driven open coding. Further, the coding was used to identify recognizable patterns of similar separation situations across the dataset and in relation to the study aim. Subthemes and themes were identified in order to obtain an idea of how they fitted and to describe the pattern of the data. In Table I, an example of the thematic structure of the data extract is shown. The themes were reviewed and discussed several times. To minimize overlap, some of the themes were merged and some of the themes were renamed o improve understanding. The thematic analysis resulted in six themes and 19 subthemes (Table II). Two themes were common to all three units, one theme was common to two units and three themes emerged in only one unit.

**Table I.** Example of the thematic structure of data extract, subthemes and themes on the labour ward

Data extract	Subthemes	Theme
Mother should be examined by a physician for suturing. The physician asked the partner to take the newborn who stayed with the partner for 20 minutes. When the suturing was completed, the newborn was placed skin to skin with the mother.	Suturing of rupture postpartum by a physician	Mother in need of examination or treatment

**Table II.** Overview of subthemes and themes

Subthemes	Themes	Origen
Tired mother or parent Protect body integrity Mother’s or parent’s own needs Feelings of fear	Mother’s or parent´s own decision	Labour ward Maternity ward, Neonatal unit
Rules and procedures as barriers Sick mother treated outside the labour or maternity ward; partner’s dilemma High workload Lack of professional understanding Lack of healthcare logistics	Deficit in organization, infrastructure, or resources	Labour ward Maternity ward, Neonatal unit
Newborn taken out of labour room Need for special medical treatment	Newborn in need of neonatal care	Labour ward Neonatal unit
Mothers on their own Handing over to the partner Old routines as barriers	Postpartum care after birth, the “Golden hour”	Labour ward
Treatment of haemorrhage or massage of uterus Suturing of rupture postpartum by a physician	Mother in need of examination or treatment	Labour ward
Complicated birth Partner’s fear of being alone with the newborn Partner not present	Mother in need of extra care	Maternity ward

Furthermore, in order to minimize the separation between mother/parent and newborn, the need for collaboration within the caregiver’s own unit, as well as between the units, was examined (Table III).

**Table III.** Need for collaboration within the caregiver’s own unit and with external units to minimize separation between parents and newborn

Themes	Labour ward	Maternity ward	Neonatal unit	Prenatal care centre	Postoperative care	Intensive care
**Labour ward**						
Mother’s or parent´s own decision	x			0		
Deficit in organization, infrastructure and resources	x					
Newborn in need of neonatal care	x		0			
Postpartum care after birth, “The Golden Hour”	x			0		
Mother in need of examination, treatment and special care	x				0	0
**Maternity ward**						
Mother’s or parent’s own decision		x		0		
Deficit in organization, infrastructure and resources		x				
Mother in need of extra care		x		0	0	0
**Neonatal unit**						
Mother’s or parent’s own decision			x	0		
Deficit in organization, infrastructure and resources			x			
Newborn in need of neonatal care	0		x			
Need for collaboration within the caregivers own unit = **x**						
Need for collaboration with external unitso =**0**						

### Ethical considerations

The head of the department gave their oral consent to the study. The observations were made on a voluntary basis by the caregivers at each unit. None of the collected data can be shown to derive from parents, newborns or caregivers involved in the study. The study complies with Swedish law relating to the ethical review of research involving humans (SFS, 2018) and the World Medical Association and Declaration of Helsinki’s principles for medical research involving human subjects, the purpose of which is to protect individuals and ensure respect for human dignity (World Medical Association [WMA], 2008)

## Results

### Themes on the labour ward

On the labour ward, zero separation was defined as skin-to-skin contact with the mother during the first two hours, followed by skin-to-skin contact with one of the parents.

Based on the analysis, five themes were constructed in which separation between mother and newborn occurred on the labour ward: *mother’s or parent’s own decision; deficit in organization, infrastructure, or resources; newborn in need of neonatal care; postpartum care after birth, “The Golden Hour”; mother in need of examination or treatment.*

#### Mother’s or parent’s own decision

Separations between mother and newborn occurred due to the mother’s own wishes. In some cases, mothers said that they needed to rest after birth. Some parents dressed the newborn directly after birth and one woman did not want to lie with a bare chest. For a no known reason, one mother did not hold her newborn close after birth.

#### Deficit in organization, infrastructure, or resources

Mothers transferred postpartum to the operating unit for placenta removal or major perineal tears were separated from their children both during the intervention and afterwards due to observation on the post-operative ward. If the mother had been under full anaesthesia, mother and newborn were separated for a longer time period. If delivered by caesarean birth, the newborn was not in a skin-to-skin position nor was it in this position during an operation with spinal anaesthesia or full anaesthesia. When the mother returned to the labour room after the operation, the newborn was positioned with the mother or the other parent with clothes on. In all caesarean births observed in full anaesthesia, healthy newborns stayed with the other parent who was clothed. Shortcomings in communication between caregivers caused a separation between mother and newborn due to the physician wanting to meet the mother before discharge from hospital at the same time as blood samples were going to be taken from the child.

#### Newborn in need of neonatal care

When newborns needed neonatal care, they were separated from their mothers, taken out of the labour room, and transferred to a special room at the delivery unit for observation and care given by neonatal caregivers. In some cases, the separation lasted for just for a short time, i.e., to check saturation or blood sampling without any need for other treatment. Sometimes, the newborn needed further care and was transferred to the neonatal unit.

#### Postpartum care after birth, “The Golden Hour”

During postpartum care, various moments of separations occurred between mother and newborn. Mothers on their own were unable to hand over their newborn in the meantime when they needed postpartum care, to go to the toilet or take a shower. On some occasions, the newborn was placed on the other parent’s chest with or without clothes within two hours postpartum. One newborn was also laid in a bed which one of the caregivers had brought from the maternity ward.

#### Mother in need of examination or treatment

Separation occurred in the delivery room because the mother needed to be examined and or treated for postpartum haemorrhage, massage of the uterus or suture of perineal tears by the physician, for example. In this case, the newborn was handed over to the other parent and returned to the mother when the examination or treatment was completed.

### Themes on the maternity ward

In the maternity ward, zero separation was defined as skin-to-skin contact with one parent or with one of the parents being present with the newborn all the time.

Based on the analysis, three themes were constructed in which separation between parents and newborn occurred on the maternity ward: *mother’s or parent’s own decision; deficit in organization, infrastructure, or resources; mother in need of extra care*.

#### Mother’s or parent’s own decision

Separations occurred when the mother asked the caregivers for help taking care of the child in situations in which the mother or both parents wanted to leave the maternity ward to smoke, visit the pharmacy, the hospital restaurant, or just go out for a walk without the newborn. Both short and long separations occurred when the newborn was transferred to the neonatal unit and the parents did not follow for unknown reasons. In some cases the mother or both parents did not want to accompany the newborn during examinations or blood samples at the maternity ward for reasons of fear or because they just wanted to rest for a while. In one case after a caesarean birth, the partner was afraid to be alone with the newborn and left the newborn with the caregivers. In one observation, the mother wanted to pump breast milk in peace.

#### Deficit in organization, infrastructure, or resources

Separation occurred when the mother was treated outside the maternity ward; observation at the post-operative department after a caesarean birth, suturing under full anaesthesia and under special care at the intensive care unit. When the partner left the maternity ward in order to stay with the mother, a separation occurred because no newborns were allowed to stay at the intensive care unit. When the newborns were enrolled at the neonatal unit for further care, several separations occurred because the parents had to wait for a family room at the neonatal unit.

#### Mother in need of extra care

Incidents of separation occurred on the maternity ward in cases where the mother had undergone a caesarean birth or after complicated labour, such as labour with severe pain and a postpartum haemorrhage. Furthermore, separations occurred for mothers with pre-eclampsia, for mothers with fever and for recently operated mothers too tired to be together with the newborn when there was a need for blood sampling in the examination room. In some of these cases, the partner was not present, and the caregivers took care of the newborn.

In various cases, parents said that they were exhausted and asked for help with premature newborns in need of extra feeding, newborns with a great need to suck or with nausea, and neonatal crying. One single mother expressed a fear of falling asleep in case something might happen to her newborn.

### Themes at the neonatal unit

On the neonatal ward, zero separation was defined as skin-to-skin contact with one parent or one of the parents being present with the newborn all the time.

Based on the analysis, three themes were constructed in which separation between mother/parents and newborn occurred on the neonatal ward: *mother’s or parent’s own decision; deficit in organization, infrastructure, or resources; newborn in need of neonatal care.*

#### Mother’s or parent’s own decision

In some of the observations, the parents wished to leave the neonatal unit together and go home to siblings. Some parents who stayed in the family room said that the sound of the alarm from the monitoring disturbed their own sleep and they wished to sleep alone. When the newborns were taken care of in the nursery room, some parents who stayed in the family room made only short visits to the newborn. The parents of one newborn wh1.o were previously enrolled at a hospital and who were used to sleeping separately from the newborn child during the night wanted to continue this routine. In one observation, the newborn was directly transferred to the neonatal unit and was separated from both its parents because the other parent who first accompanied the newborn returned shortly afterwards to the mother on the labour ward.

#### Deficit in organization, infrastructure, or resources

Different separation incidents were observed due to a deficit in organization, infrastructure, or resources. One newborn in need of special care at the maternity ward was unnecessarily transferred to the neonatal ward due to a high workload and a lack of sufficient caregivers at the maternity ward, a situation that caused separation between mother and newborn. Due to the lack of both a family room and beds on the maternity ward, one mother had to stay at a unit where newborns were not allowed. A transfer between hospitals in the region caused a separation when mother and newborn were transported separately. Furthermore, a mother with diabetes was urged to go back to the maternity ward because it was time for her to take her insulin. She had just arrived at the neonatal unit and held her newborn skin to skin for the first time. Some mothers in need of rest and partly confined to bed were only able to make short visits to their newborn child. The newborn child was then alone for most of the day. The other parent was not present at the hospital.

#### Newborn in need of neonatal care

Different separation situations occurred when newborns were transferred from the labour room to the neonatal ward for respiratory disorder and when medical interventions, i.e., catheterization, intubation, X-ray or medical care, were necessary. On these occasions, the newborns were separated from their mothers within the two hours postpartum and sometimes directly after birth. One newborn was separated from its mother and transferred to the neonatal unit for respiratory support, but on arrival the newborn did not require any support.

A further analysis of the themes revealed that there is a need for collaboration within the caregiver’s own unit but also between the units to optimize zero separation. All three units showed a need for collaboration with the prenatal care centre (Table III).

## Discussion

In this study, we have followed parents and their newborns during their hospital stay from birth to the stay on the maternity ward and in some cases at the neonatal unit. The aim of the study was to observe separation incidents between parents and their newborn child in all three units separately from one another.

At all three units, separation occurred due to clinical routines which could be traced to a lack of knowledge among caregivers and established routines created by caregivers, which were unrelated to evidence-based knowledge.

For instance, this could be seen on some occasions when the mother needed postpartum treatment or an examination on the labour ward and the newborn was handed over to the other parent. One established routine on the labour ward was to make the mother and healthy newborn ready for transfer to the postnatal ward within two hours postpartum. This was something that always caused a separation between mother and newborn.

At our hospital, newborns were routinely separated for a short time from their parents and taken to another room for a blood sample or examination directly after birth or during the stay on the maternity ward. This situation was observed on various occasions. Shortcomings in the communication between caregivers were also seen during observation. For example, one mother with diabetes was forced to go back to the maternity ward because it was time for her to take her insulin. She had just arrived at the neonatal unit and held her child skin to skin for the first time. Ballatero et al.found that practice-based norms and rules set by a specific hospital unit or facility could be a barrier to skin-to-skin contact, even though caregivers regarded skin-to-skin contact as an effective evidence-based practice intervention (Balatero et al., 2019). According to Thernström Blomqvist et al. (2013), a lack of knowledge and attitudes could be one reason why caregivers let other routines go ahead of skin-to-skin contact (Thernström Blomqvist et al., 2013).

Organizational shortcomings which reduced the opportunity for optimal skin-to-skin contact were also observed. This could be seen in all caesarean births both in the operating theatre and when the mother needed to be transferred to a postoperative unit for observation, a place newborns and partners were not allowed to enter.

Since 2009, the World Health Organization has recommended that newborns should remain with their mother immediately after birth and experience skin-to-skin contact for at least an hour, regardless of delivery method, i.e., even a caesarean birth (WHO, 2009). Despite this recommendation, there are still difficulties in the consistent implementation of skin-to-skin contact for mother and newborn after a caesarean birth (Abdulghani et al., 2018).

Barriers to the implementation of skin-to-skin contact after a caesarean birth have been reported by Alenchery et al. to depend on various factors, such as low staffing, time restrictions, lack of skin-to-skin criteria, safety concerns, adverse event risk, change in routine and a lack of collaboration (Alenchery et al., 2018; Zwedberg et al., 2015). Findings by Balatero et al. showed that providing immediate skin-to-skin contact was not regarded as a priority during the operation (Balatero et al., 2019). A study in a Swedish setting describes midwives’ experiences of mother-newborn skin-to-skin contact after a caesarean birth as: “Fighting an uphill battle”. Skin-to-skin contact is not prioritized, because many caregivers are unaware of its positive effects and their care reflects this lack of knowledge (Zwedberg et al., 2015).

Women who experienced skin-to-skin contact during a caesarean birth have described the experience as meaningful, their focus was on their newborn and they were not “aware” of the surgical procedure (Crenshaw et al., 2012; Frederick et al., 2016). The study result showed that in our hospital, there was a gap between current practice related to caesarean birth and the recommendation by the WHO, which resulted in skin-to-skin contact not occurring, sometimes for many hours. To change a current practice, collaboration and education between all the units involved in the care is required to increase caregivers’ awareness of the hospital’s skin-to-skin practice after a caesarean birth or during postpartum intensive care (Alenchery et al., 2018).

Due to a lack of both caregivers and a family room, the mother was only able to stay with her newborn for a short time. A study by Zwedberg et al. reported that a lack of effective collaboration between different wards and caregivers could interrupt skin-to-skin contact (Zwedberg et al., 2015).

Separation due to a parent’s decision or wishes could be seen at all three units after birth. A wish to put clothes on the newborn already at the labour ward or to give the newborn to the partner directly after birth was seen. At the maternity ward and at the neonatal unit, separations were observed when the parents wanted to participate in activities together and therefore left the newborn alone with the caregivers. Furthermore, separation also occurred when parents both on the maternity ward and at the neonatal unit avoided accompanying the newborn to the examination room for a blood sample. These were factors that indicated that some parents may not have understood the meaning of continuous skin-to-skin contact, something which is also underlined in earlier studies (Zwedberg et al., 2015; Dabrovski, 2007). The parents’ desire to spend time on their own was recognized as an obstacle for skin-to-skin contact in a study by Thernström Blomqvist et al. (2013). The time at the neonatal unit is not self-selected and is a vulnerable situation where one of the caregivers’ important tasks is to support the parents so they can be involved in the care of their newborn.

Birth itself is a major upheaval for both the woman giving birth and the partner, due solely to the fact of becoming parents. In a study by Kurth et al., the mothers expressed both physical tiredness, which was attributed to sleep deprivation, and emotional tiredness resulting from new impressions, concerns and challenges relating to the newborn (Kurth et al., 2010). For this reason, tiredness after birth, both physical and mental, can be due to various reasons and can be seen on the labour ward, the maternity ward and on the neonatal unit. Some women experience extreme tiredness (postpartum fatigue), which can be described as a reduction in the capacity for physical and mental activity after childbirth, a persistent lack of energy, impairments in concentration and attention which are not easily relieved by rest or sleep (Henderson et al., 2019).

In our study, separation due to tiredness occurred on several occasions on the maternity ward. Both mothers and partners said that they were exhausted for different reasons and asked for help with the newborn child. Some newborns showed an increased need for care related to neonatal crying, increased suction demand, newborns with nausea or newborns with an extra need to feed.

At our hospital, the opportunity for the partner to stay with the mother and newborn on the postnatal ward around the clock was dependent on the availability of a single room. First-time parents had precedence. In cases in which the mothers did not have a partner with her during her stay on the maternity ward, several separation incidents occurred. There is a need for caregivers to support and help mothers to provide skin-to-skin contact instead of just taking over the care of the newborn in order to give the mother some rest.

Sensitivity and professional skills are required of caregivers in order to assess the individual woman’s/parent’s need for support measures. This means balancing between encouraging and supporting skin-to-skin contact and providing care tailored to individual needs and the mother’s own choice.

Some mothers needed to stay longer than normal on the maternity ward because their state of health did not allow them to be discharged. In our study, several incidents of separation were observed on the maternity ward in these cases when the mother was ill, had undergone a caesarean birth or after complicated labour. These women had difficulty taking care of both themselves and their newborn and were in need of extra care.

The partner’s opportunity to stay on the maternity ward is seen as an important resource for both mother and newborn in providing moral support and physical care. By supporting the mother’s recovery and her ability to return to strength, the interaction between mother and newborn can be supported. According to DiMatteo et al., educational antenatal courses can help prepare partners for their empowerment and adapting to new family roles after a complicated birth (DiMatteo et al., 1996).

Key elements for successful skin-to-skin contact are raising awareness of the roles and responsibilities of caregivers, developing good communication skills, and encouraging interprofessional collaboration. This applies between the labour ward, maternity care, and the neonatal unit, together with collaboration with external caregivers at intensive and surgical care units, as well as the prenatal care centre. In this study, themes were constructed to describe incidents of separation occurring between mother/partner and newborn at the different units. Based on the themes, we are able to see that there is a need for collaboration not only within the caregiver’s own unit but also between the units in order to optimize zero separation. In the analysis, it emerged that the prenatal care centre has an important educational role to play in making parents aware of the importance of zero separation. Earlier studies have reported the importance of information about skin-to-skin contact and that woman who received information during pregnancy became more aware of the advantages of skin-to-skin contact (Alenchery et al., 2018; Thernström Blomqvist et al., 2013). Education among both caregivers and parents is essential to influence organizational and economic barriers, as well as old routines that might still exist due to attitudes and beliefs.

The main limitation of this study is that there is no documentation regarding the profession, age and professional experience. It is possible that some incidents of separation are not observed, but the observation phase lasted for four months on day, evening and night shifts. The strength is that incidents of separation were observed at all the units where the mother and the newborn stayed after birth, i.e., labour ward, maternity ward and neonatal unit.

## Conclusion

Our study shows that there is still a gap between the latest evidence-based knowledge of the importance of zero separation and current practice in newborn care. To minimize separation between parents and newborn after labour, there is a need for continuous collaboration between all three units (labour ward, neonatal unit, maternity ward) inclusive maternity clinic to create a chain of care that contributes to mother and newborn togetherness.
